# Adjustment of Couples to the Transition to Retirement: The Interplay of Intra- and Interpersonal Emotion Regulation in Daily Life

**DOI:** 10.3389/fpsyg.2021.654255

**Published:** 2021-06-18

**Authors:** Andrea B. Horn, Sarah A. Holzgang, Vanessa Rosenberger

**Affiliations:** ^1^University Research Priority Program “Dynamics of Healthy Aging,” University of Zurich, Zurich, Switzerland; ^2^Center of Competence for Gerontology, University of Zurich, Zurich, Switzerland; ^3^Psychopathology and Clinical Intervention, Department of Psychology, University of Zurich, Zurich, Switzerland; ^4^Department of Consultation-Liaison Psychiatry and Psychosomatic Medicine, University Hospital Zurich, University of Zurich, Zurich, Switzerland

**Keywords:** interpersonal emotion regulation, disclosure, transition to retirement, adjustment disorder symptoms, expressive writing, daily diary, interplay intra- and interpersonal emotion regulation

## Abstract

**Background:** Retirement is a central transition in late adulthood and requires adjustment. These processes not only affect the retired individuals but also their romantic partners. The aim of this study is to investigate the interplay of intrapersonal emotion regulation (rumination) with interpersonal regulation processes (disclosure quality). Furthermore, the associations of daily retirement-related disclosure with adjustment symptoms in disclosing and the listening partner will be investigated. It is expected that the effects of disclosure alter after providing the couples with a self-applied solitary written disclosure task in order to support their intrapersonal emotion regulation.

**Methods:** In this dyadic online-diary study, 45 couples (*N* = 45) with one partner perceiving the adjustment to a recent retirement as challenging reported rumination, perceived disclosure quality (repetitive, focused on negative content, hard to follow, disclosing partner open for common/authentic), retirement-related disclosure, and ICD-11 adjustment symptoms preoccupation and failure to adapt were assessed at the end of the day over 14 days. In the middle of this assessment period, couples performed a modified online-expressive writing about their thoughts and feelings regarding the transition to retirement.

**Results:** The double-intercept multilevel Actor–Partner Interdependence Models (APIM) reveal that on days with more daily rumination, the spouse perceived that disclosure of the retiree is more difficult to follow, more negative, and repetitive. In contrast, the retiree perceived less authenticity and openness to comments during disclosure on days when the spouse reports more rumination. Retirement-related disclosure showed no within-couple association with failure to adapt but actor effects on preoccupation. Moreover, a partner effect of disclosure of the retirees on the preoccupation of spouses could be observed. This contagious effect of the retiree disclosure, however, disappeared during the week after writing.

**Conclusion:** Our results support the notion that disclosure processes are altered during maladaptive intrapersonal emotion regulation processes. This in turn seems to lead to less effective interpersonal regulation and contagious spilling over of symptoms.

Supporting intrapersonal emotion regulation seems to have the potential to allow more favorable interpersonal regulation processes and to free interpersonal resources for an individual adjustment. This has implications for further planning of support for couples facing life transitions and aging-related changes.

## Introduction

Relationships are an important resource in life (Coan and Sbarra, 2015; Kiecolt-Glaser and Wilson, 2017). Coping with a challenge together expands the resources of the individual on the partner, and it not only activates individual resources like self-regulation and self-efficacy but also adds genuine relational processes to the regulation equation (Bodenmann, 1997; Rohrbaugh et al., 2004; Helgeson et al., 2018; Rentscher, 2019). Accordingly, romantic relationships have been identified as highly relevant when it comes to adjusting to difficult health situations (Manne et al., 2004) and life transitions like a central one in late adulthood—the transition to retirement (Havighurst et al., 1969; van Solinge and Henkens, 2005).

From a life-span perspective, it has been emphasized that retirement—which represents a change in the work sphere—is influenced and influences other life spheres—most prominently the sphere of romantic relationships (van Solinge and Henkens, 2005). Retirement adjustment has been defined as “a longitudinal process during which the levels of retirees of adjustment may fluctuate as a function of individual resources and changes in these resources.” (Wang et al., 2011; p. 207). If these processes are not successful, symptoms linked to an adjustment disorder may arise. An adjustment disorder has been defined as “emotional disturbance arising as a consequence of a significant life event” (Maercker et al., 2013; p. 381). Again, the *socio-interpersonal context* has been assumed to play a defining role in the context of stress response (Radloff, 1977; Maercker et al., 2013; Krutolewitsch et al., 2016; Lorenz et al., 2018). More specifically, *intra- and interpersonal emotion regulations* represent basic processes that are highly predictive for adjustment problems to stressful life events (DeSteno et al., 2013; Zaki and Williams, 2013; Horn and Maercker, 2015, 2016).

As it is expected, individual trajectories of the adjustment to retirement are diverse, and in most cases, they are successful (Wang, 2007; Barbosa et al., 2016). However, research in this field has identified that high-risk groups seem to be more challenged by the transition and are characterized by high-retirement anxiety rates (Wang, 2007) and mental health problems (Butterworth et al., 2006). In these studies, the predictors of successful adjustment to retirement were physical health, finances, psychological health, personality-related attributes, leisure, voluntary retirement, and social integration in general (Barbosa et al., 2016). Again, the marital relationship was discussed as one of the most important resources for a successful adjustment (Bishop and Shoemaker, 1987; van Solinge and Henkens, 2005). Even though the central role of relationships is not in question, to our knowledge, no study so far zoomed into the daily processes of couples as a resource for adjustment after the transition to retirement. Furthermore, it is no secret and well-studied that relationship processes can go array and do not help in all circumstances—they often provoke interpersonal distress particularly in late adulthood (Rook, 2003). But what are the predictors of successful co-regulation? This study aims at contributing to a better understanding of the interplay between intra- and interpersonal emotion regulation in the daily life of couples, who consider the transition to retirement as a challenge. First, we studied the interplay of daily intrapersonal emotion regulation (rumination) with the quality of attempts of interpersonal emotion regulation (perceived disclosure quality). Is maladaptive intrapersonal emotion regulation a risk factor for less successful relational regulation? Second, we investigated the association of daily disclosure with adjustment symptoms in the daily life of couples and whether this association is altered after applying an expressive writing task supporting intrapersonal emotion regulation. Does supporting intrapersonal emotion regulation result in more favorable relational adjustment processes?

The conceptual background of this study is introduced by bridging core relationship-related processes with those of intra- and interpersonal emotion regulation. Furthermore, a short introduction to the solitary written disclosure, also referred to as *expressive writing* (Pennebaker, 1997) as a way of supporting intrapersonal emotion regulation, is provided.

For a better understanding of whether relational processes are helpful or harmful when coping with a common stressor, it is recommended to consult the concepts illustrating the establishment of *relationship quality*. The establishment of intimacy has been introduced as an interactive process involving disclosure of personal relevant content that is followed by a responsive reaction by the interaction partner (Reis and Shaver, 1988). Hereby, it is crucial that this responsive reaction is perceived as such (Debrot et al., 2012). Accordingly, it has been suggested that establishing perceived responsiveness and psychological intimacy is an indirect *socio-affective pathway of emotion regulation* (Debrot et al., 2013; Horn et al., 2018). Calling against the “lone man against the element” view on emotion regulation, relationships have been interpreted as resources for the co-regulation of emotions not only in early childhood but also throughout the life span (Coan and Sbarra, 2015; Kiecolt-Glaser and Wilson, 2017). A central *inter*personal emotion regulation strategy is *disclosure* (Manne et al., 2004) or *social sharing*, which is fulfilling socio-affective needs after emotional upheavals (Rimé, 2007). Note the overlapping key role of disclosure in both areas, namely establishment of intimacy and interpersonal emotion regulation.

From an intrapersonal perspective, *emotion regulation* has been defined as processes that involve increasing, decreasing, or maintaining emotional states in terms of their quality and intensity. These processes can be automatic or controlled (Gross, 2013). There is solid evidence that adaptive intrapersonal emotion regulation is at the core of healthy functioning (DeSteno et al., 2013). Likewise, maladaptive emotion regulation represents a major transdiagnostic risk factor for mental and physical health problems (Aldao et al., 2010). Among maladaptive strategies, *rumination* has been identified as one of the most maladaptive emotion regulation strategies (Nolen-Hoeksema and Davis, 1999). The ruminative processing style is characterized by a self-focus leading to repetitive, negative thoughts (Ehring and Watkins, 2008) and an abstract, rigid processing style (Watkins and Moulds, 2005), which, ironically, is the result of trying to avoid the negative emotional content (Wenzlaff and Luxton, 2003). It is only plausible that when these ruminative negative thought circles are shared, there will be social consequences. Accordingly, earlier studies showed that ruminators benefit less from the social support (Nolen-Hoeksema and Davis, 1999), and this form of self-focus is associated with less empathic perspective-taking (Joireman and Hammersla, 2002). In general, it has been shown that avoidant emotion regulation strategies like rumination are associated with perception of reduced authenticity and likability (Butler et al., 2003) and thus spillover to the relationship.

To sum up, considering the characteristics of rumination—rigid, repetitive, and avoidant processing style—and the findings in the literature, it is expected that when ruminative thoughts are shared, the quality of self-disclosure is altered. Disclosure might be perceived as less authentic (Butler et al., 2003), more repetitive, negative, and difficult to follow (see the characteristics of ruminative thinking style; Ehring and Watkins, 2008). In line with this expectation of interpersonal effects of intrapersonal rumination, a recent line of research focuses on the interpersonal manifestation of rumination. This had been originally studied on friendship dyads in childhood and adolescence (Rose, 2002) and can be defined as rumination in dialog—disclosing the negative content in a repetitive way to close others. *Co-rumination* or *co-brooding* (Horn and Maercker, 2016) could be established as an interpersonal risk factor above and beyond intrapersonal rumination and as an important mechanism explaining the contagion of internalizing symptoms (Stone et al., 2011).

But what can be done to avoid this spilling over of the intrapersonal process into the relationship? One established minimal intervention to support intrapersonal emotion regulation and consequently improve interpersonal emotion regulation is solitary written disclosure, also referred to as *expressive writing* (Pennebaker, 1997). This is a self-applied minimal intervention that instructs individuals to write down their deepest thoughts and feelings about a stressful experience. As in interpersonal disclosure, this requires finding words for personal relevant content, own emotional responses, and thoughts. But, in contrast to social sharing situations, it neither requires a listener nor aspires for a responsive reaction. Numerous studies with different populations reveal a small, but stable effect of this minimal intervention (Frattaroli, 2006). In the literature, improving intrapersonal emotion regulation is seen as a main therapeutic mechanism (Horn and Mehl, 2004; Horn et al., 2011). Accordingly, the buffering effect of writing against maladaptive rumination has been proven in earlier studies (Sloan et al., 2008). Instead, more adaptive ways of cognitive-affective processing are supposed to be triggered which fosters the integration of the emotional event (Horn and Mehl, 2004) and helps forming a story about the emotionally arousing event (Graybeal et al., 2002). A more coherent narrative, in turn, should be easier to share. Accordingly, the social effects of expressive writing have been reported testing the assumption that expressive writing provides a “preprocessing” that improves communication and social exchange in romantic couples after challenging experiences (Lepore and Greenberg, 2002; Slatcher and Pennebaker, 2006; Baddeley and Pennebaker, 2011; Finkel et al., 2013). To conclude, solitary written disclosure is supposed to reduce rumination. Furthermore, it is supposed to improve interpersonal regulation processes mainly by helping the individual to find a coherent narrative that can be shared more easily—particularly in times of pronounced stress experiences. For the listener, in turn, it might enhance the chances to respond in a validating and understanding way to this shared story.

So far, these processes have not been investigated in the daily life of couples older than 65 years who are facing the transition to retirement. The aim of this study is twofold. First, we investigated whether that disclosure quality represents a path by which maladaptive intraindividual emotion regulation spills over to interpersonal regulation. Is daily intraindividual *rumination* associated with different perceived *disclosure quality* in the daily life of couples facing the transition to retirement? More specifically, we investigated whether on days with more rumination, disclosure of the partner is perceived as less authentic, less open for comments, more difficult to follow, more redundant, and more repeating negative topics.

Second, we investigated whether daily adjustment is associated with interpersonal emotion regulation. On days with more *retirement-related disclosure*, are there more or less adjustment symptoms in the retiree and the partner? Furthermore, we compared the week before and after the writing task—is the association between disclosure and adjustment symptoms altered after writing about the deepest thoughts and feelings regarding the transition to retirement?

## Materials and Methods

### Procedure

The GUHR study (acronym for German “dealing together with the challenges of retirement”) included different-sex couples in which at least one partner had faced retirement recently (last 24 months) and experienced the situation still as a transition. In addition, couples were required to experience the transition to retirement as an ongoing challenge. Daily Internet access and an own email address (at least one per couple) were further inclusion criteria as this was needed for the daily online questionnaires. Couples were recruited as a convenience sample from September 2015 to December 2017 *via* different channels: mailing lists, senior universities, Facebook, retirement associations, magazines, and direct contact in public spaces.

The daily diaries were performed with personalized online surveys programmed and carried out with the survey software “Unipark.” The participants received a link by email to each daily diary questionnaire. Furthermore, couples were asked not to discuss or communicate any questions and answers with their partner throughout the study duration. Following the first questionnaire, the 14-day diary survey took place at the desired time. The start of the diary survey was always on Mondays, and the following two weeks were supposed to be as representative as possible of the everyday life of the couple. For example, there were no surveys during vacation. During these two weeks, the participants answered a short daily diary questionnaire every morning after getting up and every evening before going to bed (i.e., morning and evening questionnaires). The morning survey contained questions about momentary affect and relational variables as well as sleep quality and is not part of this study. All study variables were assessed at an end of the day diary with self-reports on affect and relationship issues. Three months after the 14-days diary survey, a follow-up survey took place. During the entire study period, the participants could send an email to those conducting the study and ask questions or raise concerns. The participants received 50 CHF as compensation per couple. This study has been approved by the Ethics committee of the Faculty of Arts at the University of Zurich (No. 08042015).

### Solitary Written Disclosure: The Modified Expressive Writing Task

On the first Saturday of the diary survey, in the middle of the daily diary assessment period, the participants additionally received a link to a writing task (i.e., expressive writing), which was embedded in an online questionnaire framework. Both partners received initiations and completed the task separately and solitarily. The instruction was based on the established expressive writing paradigm and modified for the current study as follows:

“Today, I want you to write for the next 15 min about the deepest thoughts and feelings regarding the transition to retirement/the transition to retirement of your partner. (…) You might tie what you write to parts of your life that might have changed due to the new situation: How is the current situation linked to your past, your relationships with others, or who you would like to become, or to who you have been, who you would like to be, or who you are now. What has the transition to retirement meant to your relationship? What has been difficult for you as a couple? What has been positive? What would you recommend other couples facing the situation?”

### Participants

Forty-five couples (*N* = 45) were included in the analysis. Eight couples (*N* = 8) were incomplete (totally <10 entries during the diary period), and two couples (*N* = 2) were simultaneously retired after both working full hours and could thus not be included in the current analyses. This is because the distinguishable feature of the dyad (which is required for actor partner interdependence analyses, see below) was “recently retired” vs. “partner of recently retired.” In couples with two retirees (*N* = 15), the most recently retired one, the partner who was working full time as opposed to part-time before retirement, was defined as a retiree.

The average relationship durance of the couples was *M* = 31.12 years (*SD* = 13.41), most of them were married (*N* = 35), lived together (*N* = 40), and had children (*N* = 32). For the retirees, the average months since retirement was *M* = 17.49 months (*SD* = 17.1), and the median of working hours before retirement was 42 h/weeks. Further characteristics of the retired partners (e.g., education level and reasons for retirement; Floyd et al., 1992) are depicted in Table 1. From the table, it can be noted that most of the retirees had no financial concerns about their retirement, did not retire involuntarily, and reported elevated worries about their retirement, but they do not have clinically significant depression scores and were happy with their relationship.

**Table 1:** Characteristics of the recently retired partners.

		***N***	***M/SD***
Total		45	
Females/males		29/16	
Education no college degree		17	
<51 000 CHF annual income		10	
Reasons for retirement (multiple answers possible)	Too much stress at work	4	
	Physical strain at work	4	
	Disliked work	2	
	Employer suggested retirement	3	
	Employer offered incentives	3	
	Involuntary retirement	0	
	Problems with co-workers	0	
	Wanted more time with family	10	
	Wanted more leisure time	15	
	My partner wanted that I retire	1	
	I reached the official retirement age	17	
	Own health reasons	6	
	Partner health reasons	1	
	I could afford retirement financially	16	
Worries about retirement (Mean/SD)			3.61/0.89 (range 1–5)
PHQ-9 depression score (Mean/SD)			3.14/2.79 (cut-off for mild depression = 9)
DAS-4 dyadic adjustment (Mean/SD)			10.18/1.59 (range 0–16)

*Items assessing reasons for retirement and worries are taken from Floyd et al. (1992); PHQ-9: Patient Health Questionnaire (Kroenke et al., 2001), DAS-4: Dyadic Adjustment Scale short version (Sabourin et al., 2005)*.

### Measures

All measures used in the online—“end of the day”—diary included in this study are presented in the following sections. All items could be answered on a scale ranging from 0 to 4. If necessary, there were parallel versions for the retirees and their partners (separated by slashes below).

#### Daily Adjustment Disorder Symptom

The items were chosen from the standard screening questionnaire of adjustment disorder, the adjustment disorder new module (Lorenz et al., 2016) based on its item qualities explaining the symptom group and its eligibility for daily assessment. Two major symptom groups are assumed in the adjustment disorder concept of ICD-11 (International Classification of Diseases, 11. revision): first, *preoccupation*, which manifests by excessive thinking and worrying about the stressor, and second, *failure to adapt*, which is characterized by impaired daily functional status, e.g., sleep problems or role functioning. “Failure to adapt” was assessed with the following item: “Today, it was easy for me to complete the things, that had to be done (reversed).” “Preoccupation” was assessed as follows: “Today, I could not stop to think about my/my partner's transition to retirement.”

#### Retirement-related Disclosure

Here, a modified version of other studies assessing daily disclosure in couples was used (Horn et al., 2017, 2019). The item was worded as follows: “Today I talked with my partner about my thoughts and feelings regarding my/his/her retirement.”

#### Perceived Disclosure Quality

Following are the items that assessed the different facets of perceived disclosure quality: “When my partner talked today with me about positive and negative experiences …: (1) … I found it difficult to follow. (2) … He/she was very redundant. (3) … He/she repetitively came back to the same negative topics. (4) … I perceived him/her as open for comments. (5) … I perceived him/her as authentic and open.

#### Rumination

The item assessing daily rumination was taken from earlier studies (Debrot et al., 2013) and is worded as follows: “Today, I had to think again and again about the reasons for my mood and was not able to control it.”

### Analytical Strategy

**Figure 1:**
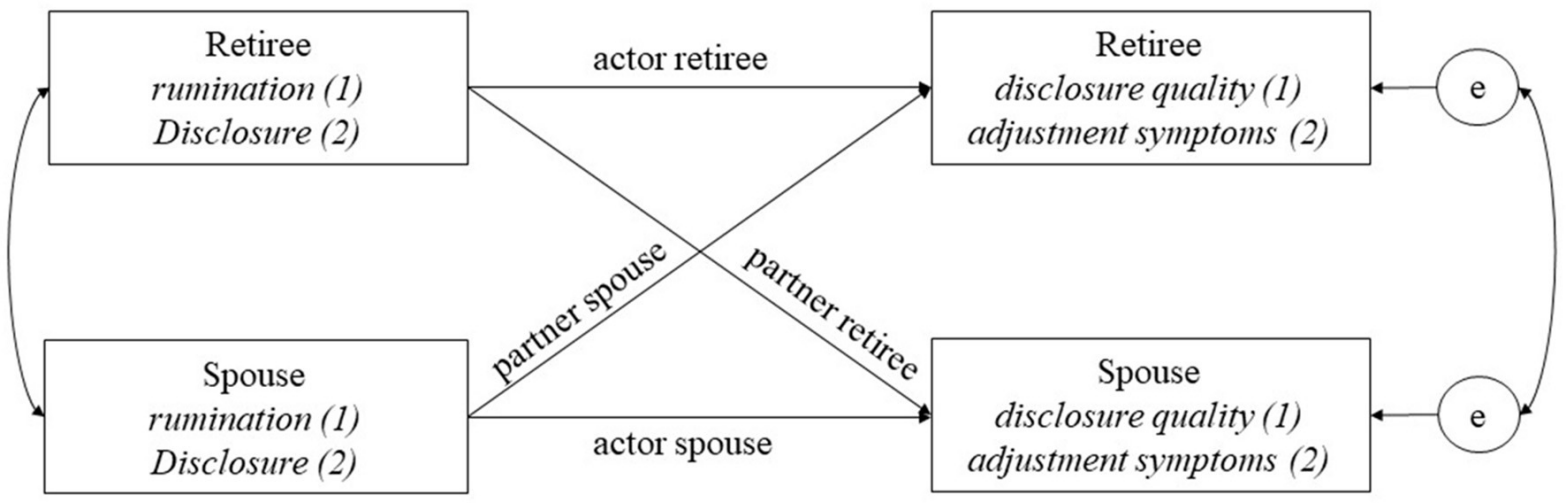
Conceptual model of this study: APIM depicting actor and partner effects that will be analyzed over time within couple. First research question: actor and partner effects of rumination on perceived disclosure quality and second research questions: actor and partner effects of retirement-related disclosure on daily adjustment disorder symptoms.

To address actor and partner effects over time, an Actor–Partner Interdependence Model (APIM; Kenny et al., 2006) was conducted within a double-intercept multilevel modeling framework for the dyadic intensive longitudinal data (Bolger and Laurenceau, 2013). APIM is a widely used analytical framework for adequately modeling the dyadic data, which allows to distinguish the effects within one partner of those crossing over to the other partner while controlling for interdependencies in the couple (Kenny et al., 2006). The APIM model of this study is depicted in Figure 1. APIMs require distinguishable dyads; in this study, the distinguishable feature was “recently retired” vs. “partner” (see also the sample description). Gender was used as a control variable in all models. In order to rely on the most parsimonious models, time centered at the middle of the assessment period and time since retirement were dropped as controls in the analyses as they did not display significant associations. All predictors were person-mean centered.

All analyses were conducted with the Mlwin software (Rabash et al., 2009). First, actor and partner effects of daily rumination on different disclosure quality measures (e.g., authentic, repeating negative content, open for comments, and redundant) were estimated in separate multilevel models controlling for gender. Second, actor and partner effects of retirement-related disclosure on adjustment symptoms (i.e., preoccupation and failure to adapt) were modeled separately. In these two models, the week before and after writing was accounted for by adding a dummy-coded predictor as well as the interaction of this variable with partner effects of retirement-related disclosure.

## Results

### Actor and Partner Effects of Daily Rumination on Perceived Disclosure Quality

**Figure 2:**
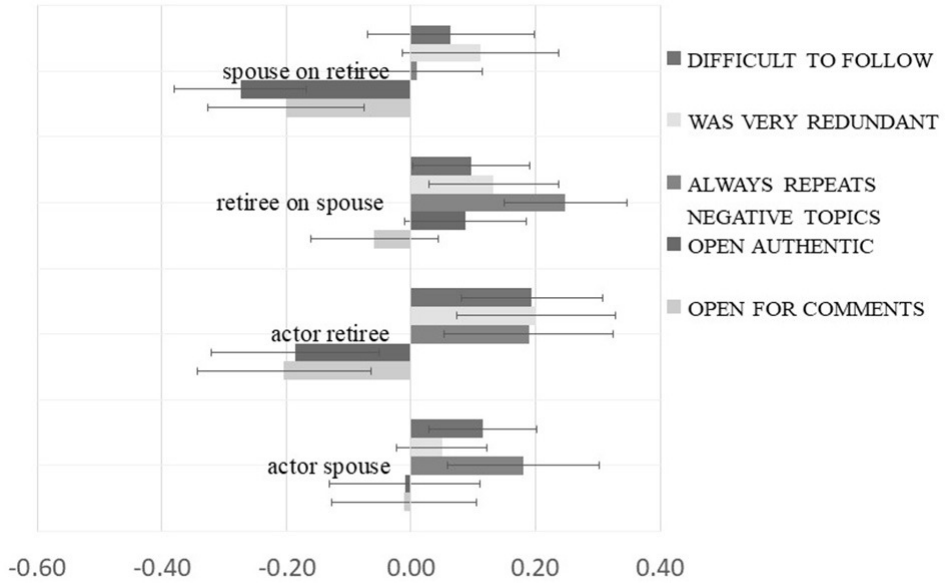
Daily rumination on perceived disclosure quality. Estimates are unstandardized betas of double intercept (retiree/partner) APIM multilevel models controlled for gender. T-lines represent 95% CIs.

An overview of the results is depicted in Figure 2, and all estimates of the APIM multilevel analyses are given in Table 2. First, actor effects of own rumination on the perception of the disclosure quality of the partner could be detected, which were slightly different depending on the role; for the spouses of the retired partners, days of more rumination were associated with the perception of the partner as less authentic. In contrast, the retirees reported higher levels of all five disclosure quality facets on days with more pronounced levels of own rumination.

**Table 2:** Multilevel models estimates: actor and partner effects of daily rumination on disclosure quality.

	**Always repeats negative topics**			**Open for comments**			**Was very redundant**			**Was authentic**			**Was difficult to follow**		
	**Estimate**	**S.E**.	**CI 95%**	**Estimate**	**S.E**.	**CI 95%**	**Estimate**	**S.E**.	**CI 95%**	**Estimate**	**S.E**.	**CI 95%**	**Estimate**	**S.E**.	**CI 95%**
**Fixed part**															
Intercept retiree	1.23	0.16	**[0.91, 1.55]**	3.41	0.16	**[3.10, 3.72]**	1.30	0.07	**[1.17, 1.43]**	3.47	0.16	**[3.15, 3.79]**	1.46	0.08	**[1.30, 1.63]**
Intercept spouse	1.22	0.20	**[0.83, 1.60]**	3.49	0.18	**[3.13, 3.84]**	1.19	0.08	**[1.04, 1.34]**	3.75	0.20	**[3.37, 4.14]**	1.32	0.09	**[1.15, 1.48]**
Actor spouse rumination	0.18	0.06	**[0.06, 0.30]**	−0.01	0.06	[−0.13, 0.10]	0.05	0.04	[−0.02, 0.12]	−0.01	0.06	[−0.13, 0.11]	0.12	0.04	**[0.03, 0.20]**
Actor retiree rumination	0.19	0.07	**[0.05, 0.32]**	−0.20	0.07	**[−0.34**, **−0.06]**	0.20	0.07	**[0.07, 0.33]**	−0.19	0.07	**[−0.32**, **−0.05**]	0.19	0.06	**[0.08, 0.31]**
Retiree rumination on spouse	0.25	0.05	**[0.15, 0.35]**	−0.06	0.05	[−0.16, 0.04]	0.13	0.05	**[0.03, 0.24]**	0.09	0.05	[−0.01, 0.19]	0.10	0.05	**[0.00, 0.19]**
Spouse rumination on retiree	0.01	0.05	[−0.10, 0.11]	−0.20	0.06	**[−0.33**, **−0.08]**	0.11	0.06	[−0.01, 0.24]	−0.27	0.05	**[−0.38**, **−0.17]**	0.06	0.07	[−0.07, 0.20]
Female (male)	−0.10	0.16	[−0.41, 0.21]	−0.20	0.14	[−0.48, 0.08]	0.05	0.07	[−0.08, 0.18]	−0.22	0.16	[−0.53, 0.09]	0.00	0.09	[−0.18, 0.18]
**Random part**															
Level: between couple															
Retiree	0.09	0.22		0.94	0.21		0.14	0.04		0.98	0.22		0.22	0.06	
Covariance retiree- spouse	0.02	0.19		0.61	0.18		0.07	0.03		0.63	0.19		0.03	0.03	
Spouse	0.06	0.25		0.96	0.22		0.13	0.03		1.12	0.25		0.09	0.03	
Level: within couple															
Variance retiree	0.21	0.06		0.64	0.05		0.24	0.02		0.75	0.06		0.39	0.03	
Variance spouse	0.28	0.06		0.63	0.05		0.23	0.02		0.76	0.06		0.46	0.03	

*N = 44 couples, 7 days, 1,128 observations in use. SE = standard error, estimates; fixed effects = non-standardized betas (range variables 0–4; all variables person-mean centered), CI 95%: confidence intervals 95%, if not including zero bold*.

When looking at the effect of the level of rumination of partners crossing over to the romantic counterpart above and beyond own levels of rumination that day, again different patterns for retirees and their spouses were observed; these effects of the partner revealed that on days with more rumination reported by the spouse, the retiree reports the perception of the spouse as being less authentic and open to comments. In contrast, spouses perceived more redundancy and repetitive negative content in the disclosure of their retired partners.

### Actor–Partner Interdependence Model Analyses on Adjustment Disorder Symptoms and Sleep Problems

All estimates of the APIM analyses investigating actor and partner effects of retirement-related disclosure on adjustment symptoms are depicted in Table 3. Within couples, failure to adapt symptoms did not show associations with retirement-related disclosure. In contrast, both partners did report more preoccupation on days when they shared retirement-related disclosure. Furthermore, there was a partner effect of retiree, but not spouse disclosure on preoccupation. In other words, on days when retirees talked more about their thoughts and feelings regarding retirement, the spouse reported more preoccupation.

**Table 3:** Multilevel model estimates: actor and partner effects of retirement-related disclosure on adjustment symptoms preoccupation and failure to adapt.

	**Preoccupation**			**Failure to adapt**		
	**Estimate**	**S.E**.	**CI 95%**	**Estimate**	**S.E**.	**CI 95% lower bound**
**Fixed part**
Intercept retiree	1.45	0.08	**[1.37, 1.60]**	3.56	0.11	**[3.45, 3.77]**
Intercept spouse	1.41	0.12	**[1.17, 1.64]**	3.77	0.11	**[3.56, 3.98]**
Actor spouse disclosure	0.19	0.03	**[0.13, 0.25]**	0.00	0.06	[−0.11, 0.11]
Partner spouse disclosure	0.03	0.05	[−0.06, 0.12]	−0.05	0.07	[−0.18, 0.08]
Actor retiree disclosure	0.22	0.05	**[0.12, 0.31]**	0.07	0.07	[−0.07, 0.20]
Partner retiree disclosure	0.26	0.06	**[0.14, 0.38]**	−0.18	0.10	[−0.38, 0.02]
Female (male)	−0.10	0.11	[−0.32, 0.12]	−0.05	0.10	[−0.24, 0.15]
Week after writing (retiree)	−0.07	0.07	[−0.20, 0.07]	0.20	0.10	[−0.01, 0.40]
Week after writing (spouse)	−0.01	0.07	[−0.14, 0.12]	−0.02	0.11	[−0.23, 0.19]
Interaction week after writing*retiree partner effect disclosure	−0.07	0.07	[−0.21, 0.07]	0.16	0.11	[−0.05, 0.36]
Interaction week after writing*spouse partner effect disclosure	−0.35	0.10	**[−0.55**, **−0.16]**	0.30	0.17	[−0.03, 0.62]
**Random part**						
Level: between couple						
Intercept retiree	0.154	0.039		0.31	0.079	
Covariance retiree-spouse	0.017	0.039		0.073	0.042	
Intercept spouse	0.307	0.073		0.119	0.039	
Level: within couple						
Variance retiree	0.262	0.017		0.572	0.037	
Variance spouse	0.227	0.015		0.681	0.045	

*N = 44 couples, 7 days, 1,014 observations in use. SE = standard error, estimates; fixed effects = non-standardized betas (range variables 0–4; all variables person-mean centered), CI 95%: confidence intervals 95%, if not including zero bold*.

**Figure 3:**
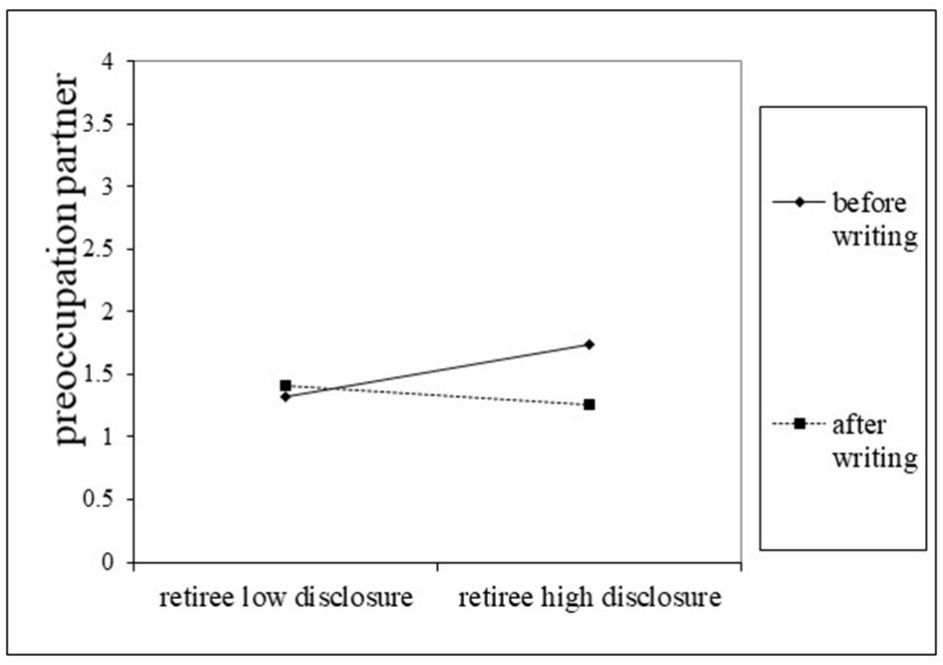
Moderation daily retiree disclosure before and after writing on partner preoccupation. High–low retiree disclosure estimates are unstandardized betas of double intercept (retiree/partner) APIM multilevel models controlled for gender and time.

There was no main effect on adjustment disorder symptoms when the week after expressive writing was compared with the prior week. However, the interaction of the partner effect of retirement-related disclosure with this dummy coded variable was significant. Figure 3 illustrates that in the week after expressive writing, the partner effect of retiree disclosure on spouse adjustment disappeared. In other words, after the writing task, days with more disclosure by their retiree were no longer days with more preoccupation with the spouse. This might suggest less spillover or possible contagion of sharing of the negative content after writing about it.

## Discussion

Summing up the main results, this study suggests the following: first, on days with more rumination, the perceived quality of disclosure in the couple was different. More specifically, disclosure was perceived as less authentic, more negative, redundant, and difficult to follow, and the disclosing partner was perceived as less open for comments. There were differential effects for retirees and their spouses; while ruminating retirees were perceived as repetitive and less open to comments, ruminating spouses were perceived as less authentic. Second, days of more failure to adapt, i.e., problems with daily functioning, did not differ in terms of couple disclosure in this study. However, partners disclosed their thoughts and feelings about retirement more on days with more preoccupation about the transition to retirement. Furthermore, we observed spilling-over effects on the partner; on days with more retirement-related disclosure by the retiree, the partner reported more preoccupation. Third, this association, however, was dampened in the week after both partners wrote about their deepest thoughts and feelings regarding the transition to retirement. More in-depth findings followed by a broader outlook, limitations, and a conclusion are discussed in the following sections.

### Perceived Disclosure Quality on Days With Intrapersonal Rumination: Actor and Partner Effects

First, we investigated whether on days with more intrapersonal rumination, perceived disclosure quality is altered. In this sample, retirees perceived disclosure of their spouses as less authentic and less open on days when the spouses reported more rumination. In contrast, the spouse perceived the ruminating recently retired partner as sharing more redundant, negative, and difficult to follow material. The differential effects of the role of partner might be explained by being more affected by the retirement transition and thus more in need of downregulating emotional responses. The qualities of disclosure of the retiree as perceived by the spouse mirror the quality of ruminative processing. So, possibly, the ruminative mode of processing the salient stressor explains the perception of the spouse. Retirees were possibly more distressed and felt an urge to share their emotionally pressing, more incoherent stories that possibly lead to co-brooding.

In contrast, the perceptions of the retiree hint at a different pattern. Here, an avoidant quality of social sharing was perceived. This might be explained by the attempt of the spouse to be supportive and understanding when talking about the retirement, which is primarily an experience of the partner and secondarily affecting the spouse. This is in line with the findings of another asymmetric situation in couples coping with the disease of one of the partners. In this context, the term protective buffering has been introduced, referring to a less authentic and less confrontative way of offering support to the ill partner with best intentions, however, mostly adverse outcomes (Coyne and Smith, 1994). Further research is needed for a better understanding of how to overcome asymmetric couple situations and maintain a sense of efficacy and autonomy in both partners which should foster positive adjustment.

The actor effects of rumination might be explained by the biased perception of disclosure of the partners associated with a ruminative self-focus. Another explanation might be that on days with more ruminative self-focus, social behavior and processing are altered and thus provoke altered disclosure quality in the partner. This way of interpreting the actor effects would be supported by findings showing less perspective-taking (Joireman and Hammersla, 2002) and less likability (Butler et al., 2003) during intrapersonal emotion regulation involving an avoidant self-focus.

### Retirement-related Disclosure and Daily Adjustment: Actor and Partner Effects

Retirement-related disclosure occurred on days with more and not fewer adjustment symptoms. Besides the lack of substantial associations with failure to adapt, preoccupation showed significant actor effects for both partners. It is important to note that these are within-person effects and reveal coupled temporal unfolding. The study results suggest that the disclosure we assessed might have been shared thoughts associated with reported preoccupation. Again, this underlines the well-documented need to share emotionally arousing experiences (Rimé, 2007). Furthermore, it supports the assumption that the quality of intrapersonal processing of emotional experiences is mirrored in the way they are shared to others. In other words, disclosure in this study might have had rather a co-ruminative nature and thus was not immediately successful in co-regulating symptoms. The partner effect of disclosure of the retirees on their spouse could be interpreted as resulting from a higher need for adjustment and emotion regulation due to the individual transition, which might lead to more incoherence and more urgency. As mentioned earlier, co-rumination is associated with more emotional contagion and maladaptive outcome (Schwartz-Mette and Rose, 2012). Further research is needed to disentangle adaptive and less adaptive ways of sharing stress-related contents and their predictors. The significant interaction results are in line with earlier findings, indicating that expressive writing has the potential to lead not only to improved intrapersonal emotion regulation but also to better interpersonal functioning. In this study, there was no placebo condition and the specific effects of writing about the retirement transition cannot be tested. Even though the interaction effect is subtle and preliminary, the spillover effect of disclosure of the retirees was dampened after expressive writing in this sample—a finding that needs to be replicated in further research and suggests an asymmetrical effect of the writing task.

### Disclosure Valence

According to the literature, there are some aspects that might be worthwhile to consider in further research, the emotional tone or the affective valence of disclosure processes being one among them. In this study, the affective valence was not assessed as we asked for thoughts and feelings regarding retirement—so it is not clear whether this rather included concerns and worries about the retirement or more enthusiastic sharing about the great new leisure time opportunities (though the latter is less probable, given the sample inclusion criterion of feeling challenged). In other lines of research investigating different forms of disclosure, the power of positive disclosure or positive sharing has been illustrated; it is supposed to allow *capitalization* upon positive experiences by sharing them and thus serving positive processes in the individual and also fostering relationship quality in couples (Gable and Reis, 2010). It has been shown that the established negative association between depressive symptom and marital quality is partly explained by reduced positive disclosure (Horn et al., 2017), hinting at the important function of daily positive disclosure for relationship quality. Furthermore, recent contributions from affective science highlight the importance of positivity resonance in relationships in general, i.e., shared moments of positive affective experiences as a driving factor for growth in personal and social resources (Frederickson, 2016). Other studies showed that mundane but not particularly emotionally loaded disclosure is highly valuable for adjustment to health problems (Robbins et al., 2018; Horn et al., 2019). The later findings are in line with the “Relational Regulation Theory”, stating the importance of interactions—including mundane but not particularly emotional ones—in everyday social interactions for successful interpersonal adjustment (Lakey and Orehek, 2011). To sum up, disclosure comes in different forms and shapes which might have differential implications for psychosocial adjustment and couple functioning. Further research considering the affective quality and intensity of the disclosed content would be of high interest.

### Aging Together

Pathways to retirement are pathways to aging, as this life transition of leaving the workforce has pronounced implications on how individuals are viewed by the society, by their romantic partner, and by themselves, and which roles they feel and are assigned to (van Solinge and Henkens, 2007; Wang et al., 2011). It is known that the value of the professional role for the self and high identification with work predicts a more demanding transition to retirement (Barbosa et al., 2016) and that there are gender effects to expect (Kim and Moen, 2002). In this study, gender could be controlled statistically, but the fact remains that most of the retirees were male and spouses being female. However, earlier studies have shown the gender differences in marital quality after retirement depending on the previous working conditions (Moen et al., 2001). This warrants further investigation with more heterogeneous samples and might lead to implications not only for including the partner in intervention supporting the individual transition to retirement (Ahlers, 2004) but also for targeting interventions to different types of couples. For example, it has been reported that dual-earner couples often plan to retire together and cohort effects on the transition to retirement reflecting different realities regarding gender equality in the workforce (Moen et al., 2006; Ho and Raymo, 2009). Other studies have found that younger dual-earner couples do not generally prefer to retire jointly, only if they report high levels of relationship and low levels of work attachment (Eismann et al., 2017) and that perceived influence on retirement decisions by the partner yields ambivalent results calling for validating the need of the retirees for autonomy while including the partner in the retirement process (Smith and Moen, 2004). Furthermore, this opens the door for further investigation into the cohort effects of the life transition and poses questions regarding possible societal changes and their effect on the transition of couples to retirement. For example, it has been proposed that as the postretirement life tends to be healthier and longer as compared with earlier generations, the individuals tend to build up a “bucket list” for the time after retirement and postpone the pursue of leisure goals into this period (Freund, 2020). This should also have an impact on the marital relationship and warrants further research. Generally, the opportunity for establishment and cultivation of leisure time activities after retirement has been discussed (Pinquart and Schindler, 2009; Zawadzki et al., 2015) which bears the potential for an increase in daily well-being (Zawadzki et al., 2015). From the perspective of a couple, this might also be worthwhile to take into account. For example, for the planning of targeted interventions, taking the advantage of establishing novel shared leisure time activity might help to open up spaces for disclosure and responsiveness and to overcome “relationship boredom” in long-term couples (Aron et al., 2000). In general, there is increasing evidence and high-conceptual plausibility that a dyadic perspective on the transition to retirement and healthy aging is indicated (Hoppmann and Gerstorf, 2009; Haase and Shiota, 2019; Horn and Röcke, 2020) and should inform future approaches investigating and supporting this transition.

### Implications and Outlook

The results of this study suggest a shifting scope in interventions. Individuals facing a life transition like retirement benefit from social resources and adaptive co-regulation in the relationship. To be successful in this endeavor, positive conditions for social sharing, in other words, good communication helps. The more possibly innovative viewpoint provoked by our findings might be that supporting individual emotion regulation also has the potential to lead to more adaptive couple processes. This is in line with earlier findings in the context of depression; in a seminal study, individual interpersonal psychotherapy showed similar effects on couple processes and depressive symptoms like an intervention focusing on enhancing dyadic coping in the relationship (Bodenmann et al., 2008). The interplay between intrapersonal repetitive thoughts and perceived disclosure quality might represent a way to analyze how intrapersonal emotion regulation deficits spillover into the relationship—possibly by verbalizing ruminative circles in dialogue. Studies conducted with adolescents in a developmental psychopathology framework support this notion: co-rumination has been shown to mediate links between depressive symptoms and interpersonal stressors over time (Hankin et al., 2010; Schwartz-Mette and Rose, 2012). It has also been reported in adult couples facing health problems—again maladaptive ways of sharing catastrophic thoughts and negative feelings about the stressor could be detected as a mediating mechanism in daily life between intrapersonal catastrophizing and fatigue symptoms after cancer (Müller et al., 2019).

Given the fact that disclosing thoughts and feelings are the starting point of the constant updating process for psychological intimacy, it does not come as a surprise that social resources might deteriorate social resources by worsening relationship quality. This is in line with earlier findings in the context of social support (Maisel and Gable, 2009), stress response (Canevello et al., 2016), enacted responsiveness (Debrot et al., 2012), and physical health (Selcuk and Ong, 2013), highlighting the importance of perceived responsiveness for positive effects on the outcome of couple-related processes. In other words, if the input of the partner comes in a context, where the romantic counterpart does not feel understood, validated, and cared for, it will not help. Sharing the overwhelming, fragmented content that has not been “preprocessed” to a coherent narrative might just be difficult to understand, and thus it is hard to transmit the feeling of being understood to the partner. Interestingly, it has been shown that the perception of being understood—not the actual level of correct understanding by the partner—is the driving force for better dyadic adjustment (Hinnekens et al., 2020). Given our findings, there might also be a risk of projecting own insecurities to the partner, a phenomenon well-studied in terms of projection of own responsiveness (Lemay et al., 2007). The non-experiential, abstract, avoidant processing mode related to rumination is opposed to functional self-reflection and self-understanding (Treynor et al., 2003), which is associated with insight into the self and all facets of positive well-being (Harrington and Loffredo, 2011). In other words, it may result in difficulty for the partner to transmit a sense of being understood to partners who do not understand themselves. It is interesting to note that this adaptive way of reflective self-focus fosters empathic perspective-taking and concern (Joireman and Hammersla, 2002) and should allow a stress expression that is easier to be answered with a responsive reaction—a script that is trained in established programs fostering dyadic coping (Leuchtmann et al., 2018). To sum up, providing alternatives for ruminative self-focus and fostering reflective self-focus not only improves adaptive intrapersonal processes but should also spillover to the relationship quality by allowing disclosure in a way that makes it possible for the partner to react in a responsive way.

### Limitations

This study has many limitations that need to be considered to prevent premature conclusions.

First, the sample size is very small. The statistical power is borderline, particularly on the couple level (level 2). With 14 points of measurement, power might be slightly more satisfying at level 1. However, replications with bigger sample sizes are warranted before relying on the findings, which furthermore reflect only small effects. This would also be important in order to strengthen the confidence in the psychometric quality of the items that have been developed for this study.

Second, given the long-recruitment period that resulted from the difficulties to find eligible couples, it is plausible to assume that the sample might be selected and does not represent all couples facing retirement or other stressors. This sample was furthermore characterized by lacking financial concerns and involuntary retirement, two factors that have been identified as risk factors for difficulties when adjusting to retirement. Furthermore, even though couples defined themselves as challenged by the transition, adjustment problems did not reach clinical significance, though they were fluctuating significantly during the assessment period. Possibly, the investigated associations do not generalize to situations when adjustment fails in a more pronounced way and leads to more severe mental health problems. To reduce the study burden and being able to recruit more burdened populations, less obstructive methods than daily diaries like mobile sensing of couple conversations in audio recordings might be possible alternatives for the future; this might allow fewer selected samples when investigating the processes. Given the very basic nature of the processes and the innovative assessment, our results are heuristically interesting and possibly inspiring future research. Furthermore, the inclusion criterion “perceiving oneself as challenged by the transition to retirement” might be vague and interpreted differently by the participating couples who had been facing the transition for different periods of time already. Further studies with a prescreening of postretirement expectancies and anxieties, current adjustment, and mental health status would allow a more specific selection of a high-risk group. Furthermore, the subjective definition “of being challenged by the transition” leads to a broad range of time passed after retirement. Therefore, it cannot be excluded that we studied couples at different stages of adjustment. Lastly, in the present study, we included only dyads who had one partner facing the transition disengaging from work life and the other partner not. However, many couples actively attempt to coordinate a joint transition to retirement which might lead to a more symmetric situation, possibly fostering less threat to self-esteem and autonomy (Zee and Bolger, 2019).

Third, this study is relying on self-reports with all their limitations. However, self-reports are the gold standard to assess perceptions (as perceived disclosure quality) and subjective experiences (e.g., rumination and adjustment symptoms). We do not know how actually disclosure sequences unfolded, what and how couples talked with each other, and what behaviors they showed. This information would add immensely to the preliminary contribution of this study and could be assessed by either inviting couples to the lab and instigating analog disclosure situations or audio-sensing daily conversation of couples (Mehl et al., 2012) and investigating their language use (Horn and Meier, 2021).

Furthermore, the reported associations are correlational in nature and do not allow causal inferences. The results reflect temporal coincidences of the study variables within couples during their daily life.

## Conclusion

The preliminary conclusion of this study is that intrapersonal risk and protective factors possibly cross over to the partner *via* interpersonal emotion regulation processes. This might play an important role when adjusting to stressors and life transitions like retirement. This study showed that disclosure in couples can also lead to maladaptive crossover effects to the partner. This is particularly the case on days when maladaptive intrapersonal emotion regulation takes place in the form of rumination. In other words, intrapersonal emotion regulation provokes changes in social life. These changes reflect mechanisms that play an important role when adjusting to stressors and have a potential impact on both partners. Improving intrapersonal regulation of emotional reactions to stressors might attenuate negative social contagion and foster adaptive sharing processes. Our findings support a socio-interpersonal perspective on adjustment to life transitions and stressful health situations and open the door for further research and interventions to support the transition to retirement and other relevant life events. A bidirectional view seems warranted—the relationship as a resource for coping better with stress but also as vulnerable to external stress influences (Lavner and Bradbury, 2017)—a vulnerability that might be prone to be overcome when considering the support of interpersonal emotion regulation.

## Data Availability Statement

The raw data supporting the conclusions of this article will be made available by the authors, upon request without undue reservation.

## Ethics Statement

The studies involving human participants were reviewed and approved by Ethics committee of the Faculty of Arts at the University of Zurich. The patients/participants provided their informed consent to participate in this study.

## Author Contributions

AH and SH conceived and designed the study. Recruitment and data collection were performed by SH and VR. Data selection and entry were supervised by AH. Statistical analyses were performed by AH. AH drafted the manuscript. All authors contributed to the writing of the manuscript and gave final approval of the version submitted.

## Conflict of Interest

The authors declare that the research was conducted in the absence of any commercial or financial relationships that could be construed as a potential conflict of interest.
